# Case of Nasal Polypus of Fourteen Years Standing, Cured by the Local Application of Peach Leaves

**Published:** 1849-07

**Authors:** E. W. H. Beck

**Affiliations:** Delphi, Indiana


### ARTICLE VII.

*Case of Nasal Polypus of fourteen years standing cured by the local application of Peach Leaves.* By E. W. H. Beck, M. D., of Delphi, Indiana.

In May, 1848, I was applied to by a man in his 76th year, but unusually stout and healthy for his age, for relief from the pressure and pain of a nasal polypus of fourteen years standing. Several times in its early stages he had permitted efforts to be made at extraction, but so tenacious and extensive were its adhesions, and so excessive the hemorrhage and pain that an entire failure was the result of each attempt. He states that it did not trouble him much till recently; at least the inconvenience was not paramount to these means of cure—and thus it continued till its enormous size and pressure urged him again to seek relief. Upon examination I found a fleshy or gelatinous tumor, occupying the whole posterior nares and extending anteriorly so as to fill nearly the whole extent of the nasal passage so that not a breath of air had passed through them in six years. The swelling was such on both sides as to produce disfiguration. As I was making a somewhat rude examination, he requested me to desist on account of the tendency of late to bleed when used roughly. Recollecting the golden rule in Prof. Mott's lecture upon this subject—never to meddle with a polypus of the hemorrhagic character, or of any kind in an old person when disposed to bleed—it was adopted. The only remedy which seemed to me to be entitled to any confidence, was the diluted hydrocyanic acid, by injection. The peach tree (amygdalus persica) was just leafing out, and knowing it contains this principle, I directed him to gather a quantity of the leaves, and after pulverizing them, to stuff them up each nostril, and to hold them in contact with the polypus by means of a wad of paper beneath and to re-apply them several times each day. In two weeks after he called on me to show me that he could do what he had not done for upwards of six years before—"blow his nose with ease," and inhale almost a sufficient quantity of air to fill his lungs with his mouth closed. He was entirely free from that pain and sense of constriction previously felt. In two weeks from this time he could sleep with his mouth closed, and in a month after, not a trace of the disease could be found. Several times since, he had, after taking cold, a sense of fulness in his nose, which soon disappears, however, on re-applying the sternutatory. He remains well and clear of it to this time.

I will only remark, in conclusion, that, in my opinion, the trial of this remedy should not be confined to this class of tumors, or to the nasal passages alone. The hydatid, carcinomatous, fungoid, and bleeding tumors, wherever situated, might yield to its deobstruent and narcotic powers.

In that varicose condition of the hemorrhoidal vessels giving orgin to hemorrhoids, and in all tumors of a similar character, I should expect much from the application of the peach leaf. Now that the attention of the profession has been directed to the subject, it is hoped that further experiments with this remedy will be made and reported.

